# Editorial: Epigenetic regulation and therapy resistance in cancer

**DOI:** 10.3389/fphar.2022.1119073

**Published:** 2023-01-09

**Authors:** Yongxia Zhu, Fabio Pittella-Silva, Yuxi Wang, Ting Wang, Fangyi Long

**Affiliations:** ^1^ Department of Pharmacy, Sichuan Cancer Hospital & Institution, Sichuan Cancer Center, School of Medicine, University of Electronic Science and Technology of China, Chengdu, China; ^2^ Laboratory of Molecular Pathology of Cancer, Faculty of Health Sciences and Medicine, University of Brasilia, Brasilia, Brazil; ^3^ Targeted Tracer Research and Development Laboratory, Institute of Respiratory Health, Frontiers Science Center for Disease-Related Molecular Network, West China Hospital, Sichuan University, Chengdu, China; ^4^ Department of Clinical Research, Sichuan Cancer Hospital & Institution, Sichuan Cancer Center, School of Medicine, University of Electronic Science and Technology of China, Chengdu, China; ^5^ Laboratory Medicine Center, Sichuan Provincial Maternity and Child Health Care Hospital, Affiliated Women’s and Children’s Hospital of Chengdu Medical College, Chengdu Medical College, Chengdu, China

**Keywords:** epigenetics, methyltransferases, RNA modifications, cancer therapy, chemoresistance

Epigenetics, defined as non-genetic modification, has become an important Research Topic in oncology. It is reported that various epigenetic modifications have occurred in cancer, such as DNA methylation, RNA modification, histone modification and other post-transcriptional regulation. Based on these findings, more in-depth epigenetic alterations or mechanism studies are expected to provide better therapies for cancer treatment (Bates, 2020). In addition, resistance to treatment severely weakens the efficacy of cancer therapy, leading to the recurrence and metastasis in various cancers. Therefore, it is crucial to explore the epigenetic regulation in cancer and the molecular mechanisms of cancer cells acquiring drug resistance and radio-resistance.

The Research Topic entitled “*Epigenetic regulation and therapy resistance in cancer*” was opened from 17 May 2022 to 18 October 2022, aiming to discuss the latest advances in epigenetic regulation in cancer, and the molecular mechanisms leading to therapeutic resistance. Finally, a total of 4 articles (3 reviews and 1 original research) with more than 30 authors contributing as experts in the field were accepted in 15 submissions, providing novel and extensive insights for future anti-cancer therapy by targeting epigenetic regulation.

As one of the most important epigenetic regulatory factors, EZH2 participates in the pathogenesis of cancer through methylation of histone and non-histone substrates or through the formation of large protein complexes (Kim and Roberts, 2016). EZH2 has become an attractive target for the development of cancer treatment in recent years. For example, a novel potent selective EZH2 inhibitor named *Tazemetostat* has just been approved by FDA in 2020 (Hoy, 2020; Slater, 2020). The original article presented by Zhang et al. reveals the effects of EZH2 on epigenetic silencing of ATG5 in gastric cancer (GC). Firstly, the results demonstrate that lncRNA LINC-PINT is downregulated in human GC tissues and cisplatin (DDP)-resistant GC cells. LINC-PINT inhibits the proliferation, migration and invasion ability of DDP-resistant GC cells and, at the same time, enhances DDP sensitivity by attenuating cell autophagy. The authors revealed that LINC-PINT recruits EZH2 to the promoter region of ATG5 to inhibit its transcription, thereby inhibiting cell autophagy and enhancing DDP sensitivity. This data further evidences a key role for EZH2, suggesting it as an attractive target in gastric cancers.

Zhao et al. discuss the role of another lysine methyltransferase, SETDB1, in the regulation of tumorigenesis and progression, immune evasion and immune checkpoint blockade resistance. The authors point out that SETDB1-targeted therapy remains challenging due to potential side effects and the non-specificity of current available inhibitors. However, the authors still hold a positive attitude towards SETDB1-targeted therapy, which may be a promising anti-tumor epigenetic therapy.

Another important Research Topic of epigenetics implicates the role of RNA modification as a key post-transcriptional regulator in cancer. RNA modifications have recently become attractive targets for cancer therapy (Barbieri and Kouzarides, 2020). In this Research Topic, Feng et al. summarize a variety of RNA modifications, including N6-methyladenosine (m6A), N7-methylguanosine (m7G), and 5-methylcytosine (m5C), N1-methyladenosine (m1A), N3-methylcytosine (m3C), and pseudouridine (ψ), and their regulatory roles in hepatocellular carcinoma (HCC). The key roles of RNA modifications in the occurrence and development of HCC provide new strategies and promising therapeutic targets for HCC therapy. However, the authors point out that there is still no suitable sequencing technology to simultaneously detect all RNA modifications in the same transcript. Eventually, the diversity of RNA modification gives us hope that RNA epigenetics will make more breakthroughs in cancer therapy.

Finally, DNA methylation mediated by DNA methyltransferase is an important epigenetic process that regulates mammalian gene expression. The changes of DNA methylation in cancer are considered as promising targets for developing powerful biomarkers for diagnosis, prognosis and prediction (Koch et al., 2018). Zhang et al. discuss the mechanisms of DNA methylation in tumorigenesis and cancer progression, and examine the recent progress and pharmacological properties of DNA methyltransferase inhibitors (DNMTi) as anti-cancer drugs in clinical and pre-clinical research. The review also points out the shortcomings of existing DNMTi, which may hinder the development of new selective DNMTi. In addition, the authors predict that the development of DNMTi will focus on non-nucleoside selective DNMTi, not only as potential therapeutic agents, but also as useful chemical probes, which will help to understand the epigenetic regulation mechanisms of DNA methylation in cancer.

In summary, the Research Topic “*Epigenetic regulation and therapy resistance in cancer*” has collected studies focusing on finding the molecular mechanisms of therapeutic resistance and summarizing the latest progress in epigenetic and post-transcriptional regulation of cancer. We hope that this Research Topic will contribute to understanding the molecular mechanisms of tumor development, and provide novel and broad insights for future anti-cancer treatment.
